# Water Temperature Observation by Coastal Acoustic Tomography in Artificial Upwelling Area

**DOI:** 10.3390/s19122655

**Published:** 2019-06-12

**Authors:** Haocai Huang, Yong Guo, Zhikun Wang, Yun Shen, Yan Wei

**Affiliations:** 1 Ocean College, Zhejiang University, Zhoushan 316021, China; hchuang@zju.edu.cn (H.H.); guo_yong@zju.edu.cn (Y.G.); 11834011@zju.edu.cn (Z.W.); shenyun@zju.edu.cn (Y.S.); 2 Laboratory for Marine Geology, Qingdao National Laboratory for Marine Science and Technology, Qingdao 266061, China

**Keywords:** artificial upwelling, costal acoustic tomography, temperature field, underwater observation, inversion method

## Abstract

Artificial upwelling is a geoengineering method to repair and improve marine ecosystems, and its operation requires long-term and continuous temperature field observation. However, existing methods are rarely seen to accomplish such observation. In this study, we investigate the coastal acoustic tomography (CAT) to obtain the long-term horizontal temperature field of an artificial upwelling area in an anechoic tank. We conduct four sets of experiments with different CAT station numbers and compare their data with those collected from temperature sensors. By analyzing the travel time from the CAT experiments, the horizontal temperature field of the upwelling area could be mapped. The CAT results and the comparison results show that the surface temperature of the observed area decreases by approximately 3 °C after upwelling, while the temperature of where the CAT is deployed decreases by about 1 °C; the temperature is lowest at the center of the upwelling area. Increasing the number of stations and station spacing would improve the temperature mapping accuracy. Therefore, the feasibility of using the CAT system to observe artificial upwelling is proved valid. This study indicates the potential application of CAT in temperature field observation in artificial upwelling area in the sea.

## 1. Introduction

Currently, more than half of the world’s population lives within 60 km from a coast. The destruction of the marine ecosystem continues to worsen owing to increased activities, such as fisheries, mineral and energy extraction, coastal development, and marine waste emissions [1]. Consequently, the use of artificial methods to repair and improve marine ecosystems has become a crucial and necessary remedy.

Artificial upwelling is a geoengineering method that artificially lifts deep seawater to the surface using pump equipment in the operating sea area. Besides changing nutrient distribution and the nitrogen and phosphorus levels, it can enhance fish stocks [2] and improve regional carbon sink capacity through efficient absorption of CO_2_ in the atmosphere [3]. It may also help mitigate local environmental extremes [4]. The optimal operation of an artificial upwelling system requires a supporting observation system; research to establish this is under way [5]. Koutaro et al. from Saga University and Tohoku University, Japan, used tracer detection systems for continuous measurement of upwelling [6]. This method could only observe the upwelling velocity of a fixed point and could not measure temperature changes at an arbitrary position of upwelling area. Xiamen University, Zhejiang University, China Ocean University, and several other universities built an artificial upwelling demonstration area in Ao Shan Bay, Qingdao, China in September 2017, with the goal of establishing a system that allows a wide range of real-time observation over a long term [7]. They used different kinds of sensors to obtain a variety of hydrological data in real-time such as water quality, conductivity, temperature, and depth(through CTD), dissolved oxygen, and pH. While observing a certain point in artificial upwelling area was possible, their study was not able to perform two-dimensional observation to obtain the data distribution in a particular region.

As an application of ocean acoustic tomography (OAT) in coastal areas, coastal acoustic tomography (CAT) has emerged as a new observation technology [8,9]. It could achieve a large range of velocity and temperature field observations and meet the long-term, real-time requirements of the field observations needed [10,11,12,13,14]. Underwater Wireless Sensor Network (WSN) can also achieve temperature field observations. It requires large numbers of sensors in order to provide environmental sampling. This indicates that deployment of WSN would incur more time and possibly higher costs. Moreover, these mobile sensors can easily float along with ocean currents and can hardly observe a fixed area, such as an upwelling area [15]. Thermocouples, resistance temperature devices (RTD), capacitance thermometers, etc., are all unable to obtain temperature field observations, and infrared thermography requires optical solutions which are not preferred, due to their short transmission distance underwater [16]. Acoustic thermography could detect changes in deep-ocean temperature, but depend heavily on an unusually stable acoustic feature in deep-ocean propagation, which can hardly be used at an offshore artificial upwelling area [17]. Overall, the CAT method is able to obtain the temperature field of an artificial upwelling area and observe the temperature distribution and the temperature changes of such areas, which is difficult to achieve using other observation methods. Some researchers have previously conducted CAT-based upwelling observation studies. Carriere from Free University of Brussels observed local coastal upwelling in Brazil’s Cabo Frio region in 2009, assimilating the obtained acoustic data into a feature model that continuously updated the prediction of the temperature field of the upwelling. The results demonstrated that the continuous tracking of the upwelling condition with the Set Kalman filter (EnKF) was better than that of the extended Kalman filter (EKF) [18]. Zhang et al., from Hiroshima University, used CAT to observe the upwelling caused by typhoons at Hiroshima Bay in 2013, and successfully obtained results consisting of temperature field, flow field, and salinity distribution of the observed area [19,20]. However, none of the above experimental observation targets relate to artificial upwelling. Additionally, they rarely perform the observation of upwelling in a small region.

In this study, we investigate CAT data to obtain the long-term horizontal temperature field of an artificial upwelling area in an anechoic tank. The temperature field distribution and its variation trend could be observed. This proves that it is feasible to apply CAT to observe the temperature changes caused by artificial upwelling. To further verify it, the data collected from temperature sensors deployed in the tank are compared with corresponding results obtained by CAT.

The rest of the paper is structured as follows. In Section 2, the relationship between travel time and temperature is established, and the inversion method is introduced to map the temperature field. Section 3 illustrates the experiment setup and describes the upwelling process in detail. Section 4 displays the data cross-correlation results and the horizontal temperature fields and compares the CAT results with the temperature sensor results. The concluding remarks are given in Section 5.

## 2. Theoretical Analysis

This section describes the mathematic methods used to obtain the temperature field. Section 2.1 establishes the relationship between travel time and temperature. Section 2.2 introduces the inversion method to map the temperature field.

**Figure 1:**
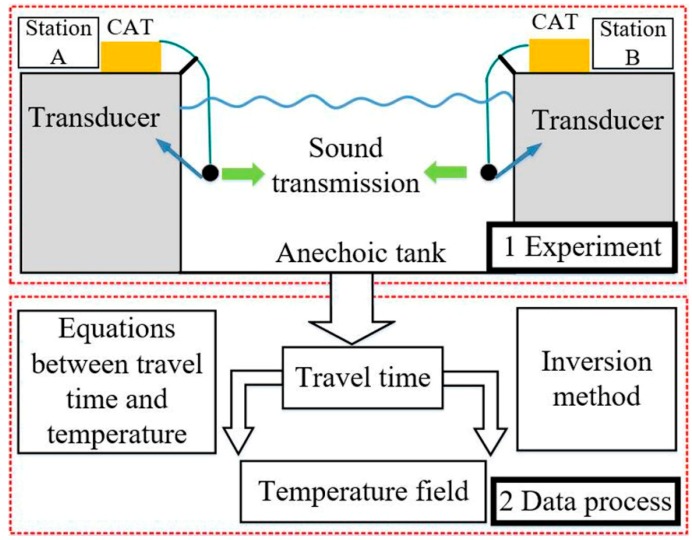
Field observation process using coastal acoustic tomography CAT.

The whole process of using CAT to observe the temperature field is demonstrated in Figure 1. The CAT instruments equipped with a transducer are first installed in the observed area in an anechoic tank. Once the experiment begins, the instruments would send each other the M sequence acoustic signal, and the travel time can be obtained after cross-correlation. At the end of the experiment, the data from each instrument are collected and the relationship between travel time and temperature is established. Subsequently, the temperature field is mapped using the inversion method. Note that the propagation of sound in a small area (no more than 100 m of station spacing in this study) can be approximated as linear propagation. Hence, the length of the acoustic propagation path can be assumed to be equal to the station spacing, and the length of the direct path need not be calculated by ray-tracing simulation.

### 2.1. Equations for Sound Speed Deviation Fields

**Figure 2:**
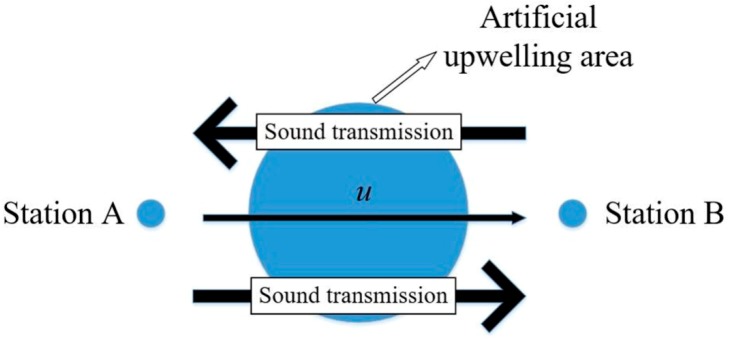
Diagram of reciprocal sound waves.

When sound travels between two points in water, it is affected by the flow velocity and the temperature of the travel path, resulting in different travel times in different directions. As shown in Figure 2, points A and B are two observation sites, while *u* is the flow component along the direction of the straight line (extending from A to B). The travel time can be obtained as follows:

(1) $$t=\oint \frac{ds}{C_{0}+\Delta C+v*n}$$

The equation depends on the underwater reference sound speed ($C_{0}$), the flow velocity (v), the unit vector along the path direction (n), and the change of sound velocity caused by temperature variation ΔC. With the constant temperature and the flow velocity of 0, we can simplify the formula as follows:

(2) $$t_{0}=\int \frac{ds}{C_{0}}$$

When $\Delta C\ll C_{0}$, $u=v*n\ll C_{0}$, the Taylor expansion of Equation (1) can be expressed as follows:

(3) $$t=\int \frac{ds}{C_{0}\left(1+\frac{\Delta C+v*n}{C_{0}}\right)}=\int \frac{ds}{C_{0}}\left(1-\frac{\Delta C+v*n}{C_{0}}+\frac{{\left(\Delta C+v*n\right)}^{2}}{{C_{0}}^{2}}-\cdots \right)$$

Ignoring the higher-order terms, i.e., exponents of 2 or higher, we obtain the following:

(4) $$t=\int \frac{ds}{C_{0}}\left(1-\frac{\Delta C+v*n}{C_{0}}\right)$$

Combining Equations (2) and (4), we obtain the following:

(5) $$\tau^{\pm }=t-t_{0}=-\int \frac{\Delta C\pm v*n}{{C_{0}}^{2}}ds$$

Since this study requiresthe temperature calculation, it is necessary to eliminate parameters that are irrelevantto the temperature. The resulting equation is as follows:

(6) $$\delta \tau =\tau^{+}+\tau^{-}=-2\int \frac{\Delta C}{{C_{0}}^{2}}ds$$

The Fourier expansion formula of ΔC can be expressed as [21]:

(7) $$\begin{array}{l} \Delta C(x,y)=a+\sum_{k=0}^{N_{x}}\sum_{l=0}^{N_{y}}\{A_{k,l}\cos2\pi \left(\frac{kx}{L_{x}}+\frac{ly}{L_{y}}\right)+B_{k,l}\sin2\pi \left(\frac{kx}{L_{x}}+\frac{ly}{L_{y}}\right)\}\\ =\sum_{j=1}^{\left(N_{x}+1)(N_{y}+1\right)}D_{j}Q_{j}(x,y) \end{array}$$

where:

$$D=\{D_{j}\}=\{a,A_{00},B_{00},A_{01},B_{01},\cdots \cdots ,A_{NxNy}\}$$

$$Q(x,y)=\left\{Q_{j}\right\}=\left\{1,1,0,\cos\frac{2\pi y}{L_{y}},\sin\frac{2\pi y}{L_{y}},\cdots \cdots ,\cos2\pi \left(\frac{N_{x}x}{L_{x}}+\frac{N_{y}y}{L_{y}}\right),\sin2\pi \left(\frac{N_{x}x}{L_{x}}+\frac{N_{y}y}{L_{y}}\right)\right\}$$

where x, y represents the two-dimensional position coordinates, and $L_{x}$, $L_{y}$ represents the length and width of the compute domain, respectively. Substituting Equation (7) into Equation (6), we obtain the following:

(8) $$\delta \tau_{i}=-\frac{2}{{C_{0}}^{2}}\sum_{j=1}^{\left(N_{x}+1)(N_{y}+1\right)}D_{j}\int_{0}^{L_{i}}Q_{j}ds$$

Equation (8) establishes the relationship between δτ and the sound velocity deviation ΔC. By solving this equation, the sound velocity in the calculated domain can be obtained. The corresponding temperature field can be obtained by substituting it into the empirical formula of sound velocity which was proposed by Medwin in 1975 [22]. This empirical formula is as follows:

(9) $$C=1449.2+4.6T-0.055T^{2}+0.00029T^{3}+(1.34-0.010T)(S-35)+0.016Z$$

In this equation, C indicates the speed of sound, while T, S, and Z represent temperature, salinity, and depth, respectively. Owing to the small size of the experimental area in this study, the acoustic signal path is approximated as a straight line, and therefore the change of depth item can be ignored. Additionally, the salinity is considered to be zero.

### 2.2. Inversion Process

The matrix form of Equation (7) is as follows:

(10) y=Ex+n

In this equation, $y=\{\delta \tau_{i}\}$ represents the time information obtained from the experiment, $x=\{D_{j}\}$ represents the unknown coefficient matrix to be calculated, $n=\{n_{j}\}$ represents the error, and $E=\{-\frac{2}{{C_{0}}^{2}}\sum_{j=1}^{\left(N_{x}+1)(N_{y}+1\right)}\int_{0}^{L_{i}}Q_{j}ds\}$ represents the known coefficient matrix. The solution to Equation (10) involves an inversion process, and it is necessary to obtain the minimum error of x under the condition where a stochastic error of n is present. The conical least squares method can be used to solve this problem. The cost function is represented as:

(11) $$J=n^{T}n+\alpha^{2}x^{T}x={\left(y-Ex\right)}^{T}(y-Ex)+\alpha^{2}x^{T}x$$

By setting the derivative of x to 0, we can obtain the value of variable x that minimizes the cost function:

(12) $$\hat{x}={\left(E^{T}E+\alpha^{2}I\right)}^{-1}E^{T}y$$

Errors can be expressed as:

(13) $$\hat{n}=y-Ex=\{I-E{\left(E^{T}E+\alpha^{2}I\right)}^{-1}E^{T}\}y$$

The coefficient α can be calculated using the L-curve method [23]. This makes a trade-off between solution and error.

## 3. Experiments

In this section, the experiment setup is introduced first, including the parameters of CAT instruments, the station layouts, and the experiment environment. After that, the method of generating upwelling is elaborated upon and the experiment processes are detailed.

**Figure 3:**
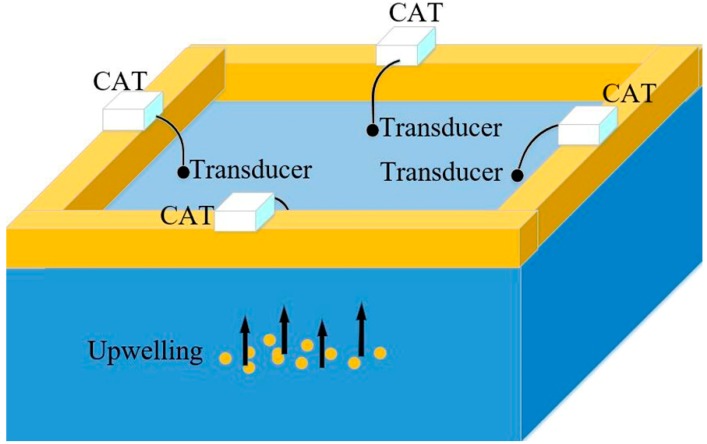
Experiment arrangement.

**Figure 4:**
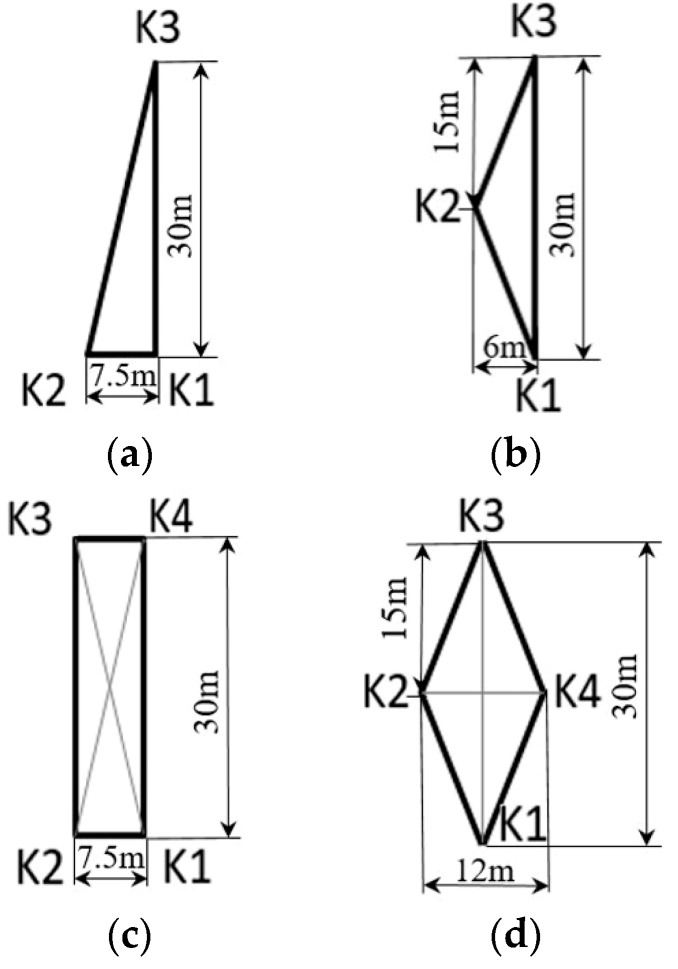
Four different station layouts. (**a**) Right triangle layout; (**b**) Isosceles triangle layout; (**c**) Rectangular layout; (**d**) Lozenge layout.

Figure 3 is the experimental layout in the anechoic tank. The upwelling device is positioned in the center of the observed area. Four CAT instruments are placed at each side of the tank. Each is attached with a GPS receiver for time synchronization and a dual-use transducer with a frequency of 50 kHz. The CAT instrument and transducer were powered by 12 V and 24 V DC power supplies respectively. The transducer was placed at a water depth of 1 m. We believed that the station numbers and station distances possibly affected the CAT observation results. Right triangle, isosceles triangle, rectangle and lozenge are considered four typical shapes, therefore, four groups of experiments with different station layouts were designed and carried out, as displayed in Figure 4. The station distances were mainly determined by the dimensions of the experimental area in the anechoic tank.

**Figure 5:**
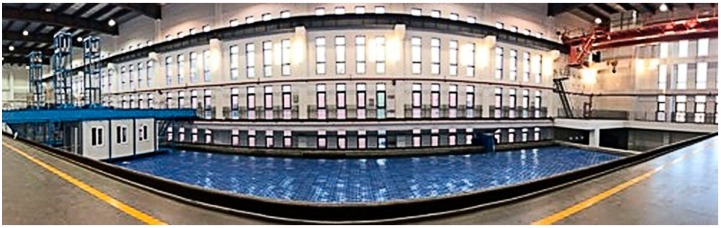
Anechoic tank.

The experiments were conducted in the anechoic tank at Zhejiang University, as shown in Figure 5. The anechoic tank, with a length of 50 m, width of 15 m, and depth of 10 m, was used to simulate a marine sound environment, and was free from disturbances to measure a variety of acoustic parameters. Its band coverage is between 1–100 kHz. A sliding experimental platform was installed above the anechoic tank. Thus, it allowed the release of the transducer, positioned the station accurately, and helped in acquiring the experimental data.

**Figure 6:**
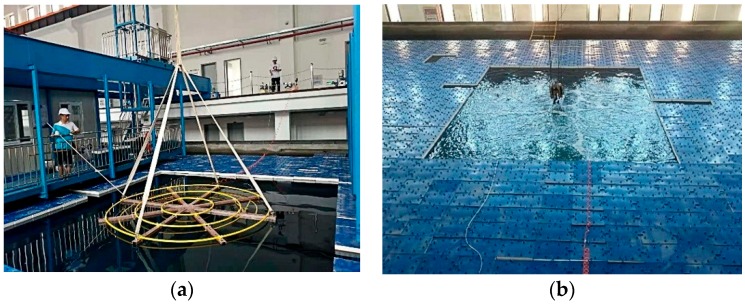
Gas injection frame layout. (**a**) Gas injection frame; (**b**) Artificial upwelling.

In this experiment, the pneumatic lifting method was used to generate the upwelling. The same method was used in the upwelling experiments conducted by Zhejiang University in QiandaoLake, China in October 2011 and December 2012 [24,25]. As displayed in Figure 6a, we designed a gas injection frame consisting of three concentric water pipe rings with a maximum diameter of 3 m. The pipe rings were made of irregular copolymer polypropylene. The gas injection frame had 90 holes that were evenly distributed with a 4.8 mm diameter. The two inlets of the gas injection frame were connected with two air compressors, to enable underwater gas injection. Before the experiment, a rectangular area of 4 × 5 m is prepared on the surface of the water, and the gas injection frame is placed 4 m underneath with the suspended lifting arm installed on the experimental platform. The upwelling is shown in Figure 6b.

The experiment lasted for 28 days, from 17 June 2018 to 14 July 2018. Each time, the CAT instruments were first placed in the still water and worked for 4 h. Then, the air compressors were switched on and the upwelling was created and lasted for 4 h. The transducer could not receive and send a signal at the same time; therefore, each instrument was only allowed to send a signal once per cycle and the time interval between signal-sending was 1 min. For example, for the stations with the right triangle layout, K1, K2 and K3 took turns sending signals with 1 min intervals, and the whole cycle took 3 min. As the reflected signal was absorbed in the anechoic tank, any multipath effect was eliminated, resulting in a high quality of the received signals. The 7-order M sequence as the acoustic signal was used and this M sequence was repetitively sent 8 times every one minute. These 8 repeated signals were averaged in data processing to improve the signal-to-noise ratio (SNR). Each code element of the sequence utilized two carrier cycles. After collecting the received data from instruments, we could obtain the travel time between each pair of stations by cross-correlation. Then the method described in Section 2 could be used for data processing to obtain the temperature field.

## 4. Results and Discussions

In this section, we first perform the signal cross-correlation to acquire the travel time. Based on this, Section 4.2 maps the horizontal temperature field with the inversion method. In Section 4.3, we compare the CAT results with the data collected by temperature sensors that are employed at different water depths. Section 4.4 discusses the accuracy of the CAT observation results

### 4.1. The Signal Correlation

**Figure 7:**
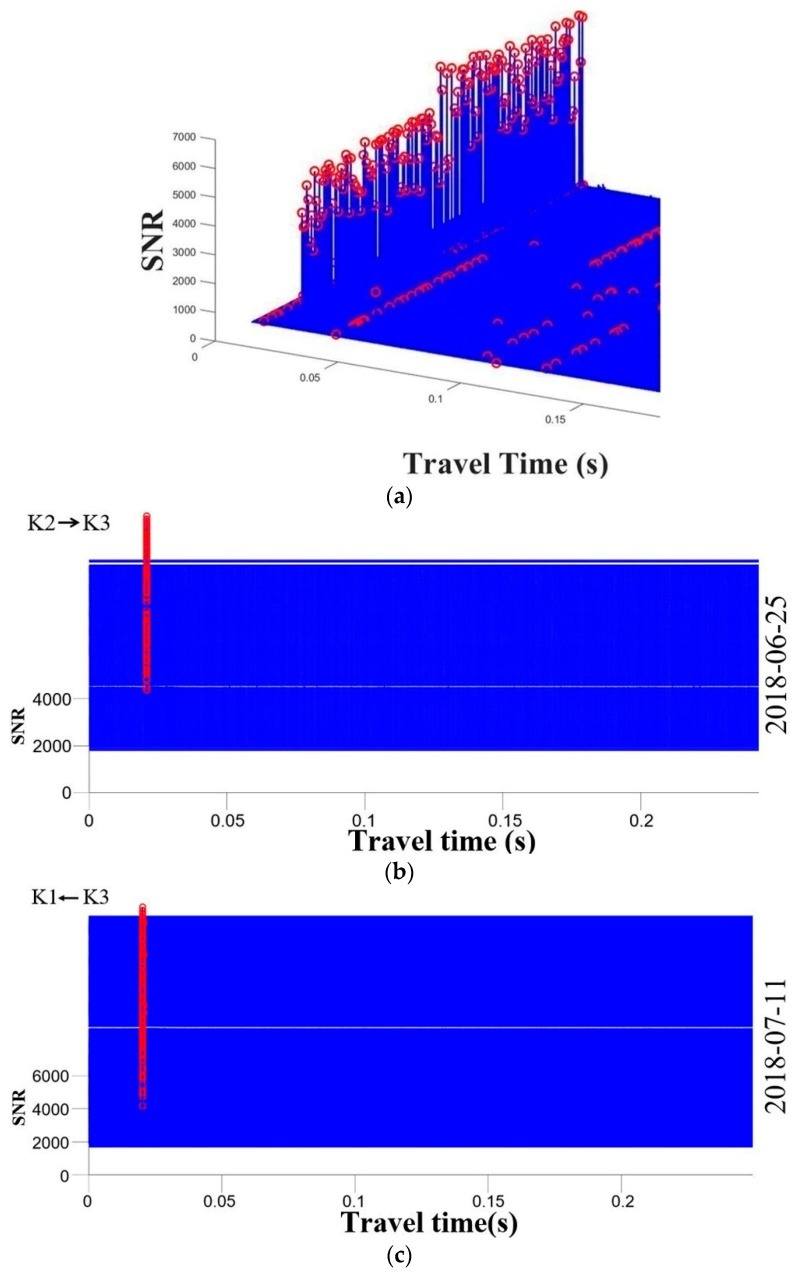

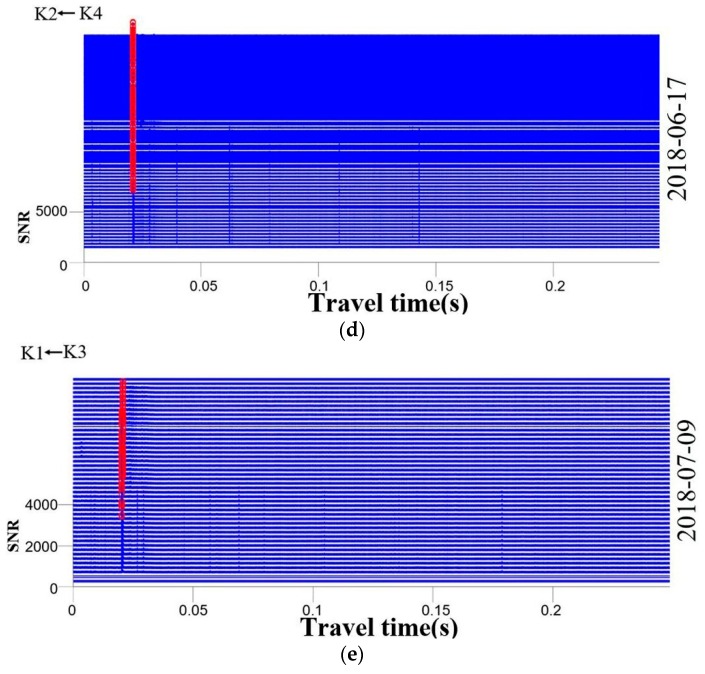
Correlation data. (**a**) Three-dimensional plot of travel time for the right triangle layout; (**b**) Travel time for the right triangle layout; (**c**) Travel time for isosceles the triangle layout; (**d**) Travel time for the rectangular layout; (**e**) Travel time for the lozenge layout.

The signal correlation is performed to obtain travel time from the collected data in the experiment. Figure 7 displays the travel time for different layouts after cross-correlation. We take a three-dimensional plot of travel time for the right triangle layout as an example (shown in Figure 7a) to explain. The red circle is the point with the highest SNR for each set of acoustic signals. The SNR of the correlation peak is significantly higher than the other positions, and the travel time can be accurately identified. As shown in Figure 7b–e, the travel time for different layouts could be obtained after eliminating the wrong circles. In addition, the number of signals in Figure 7e is significantly lower, possibly due to a weak indoor GPS signal. This might lead to the CAT instruments becoming desynchronized and further the loss of data. Overall, the quality of the collected data was regarded satisfactory and sufficient for subsequent data processing.

### 4.2. Horizontal Temperature Field Mapping

**Figure 8:**
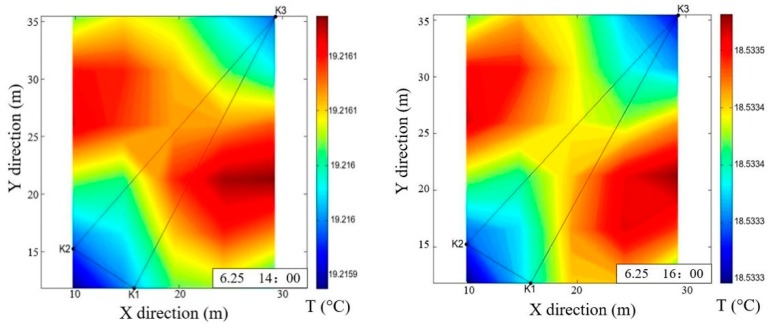

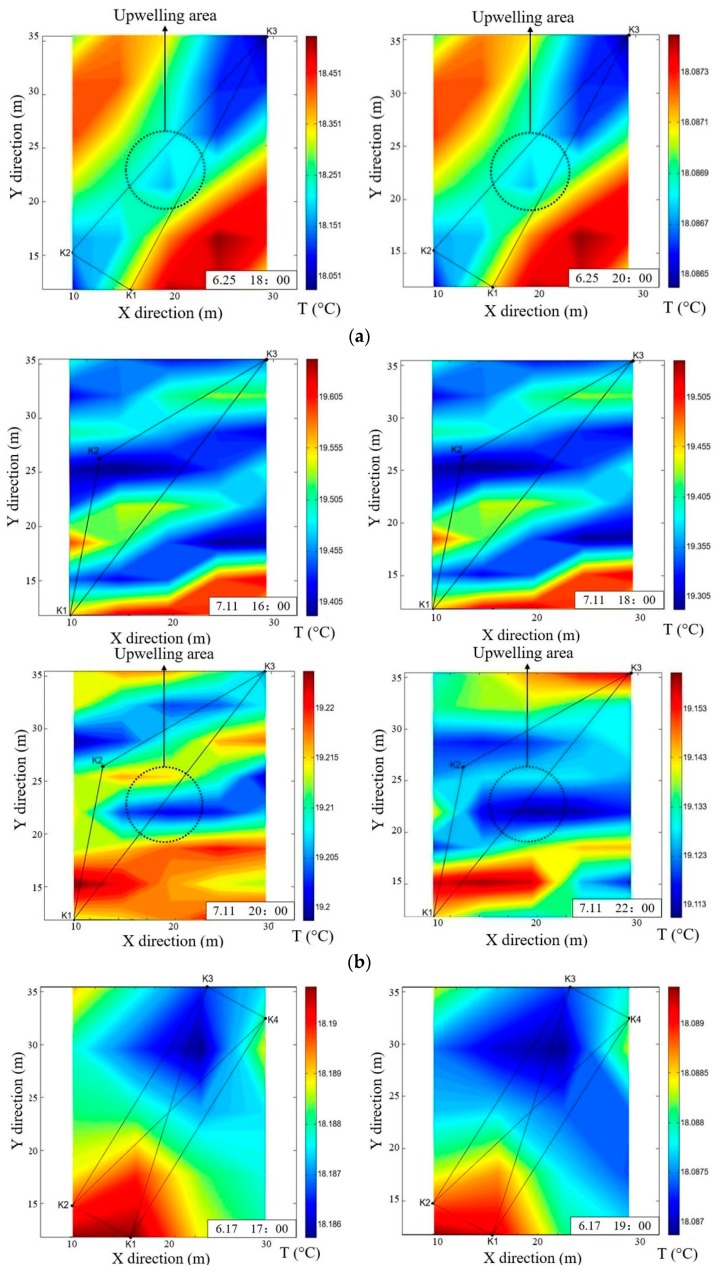

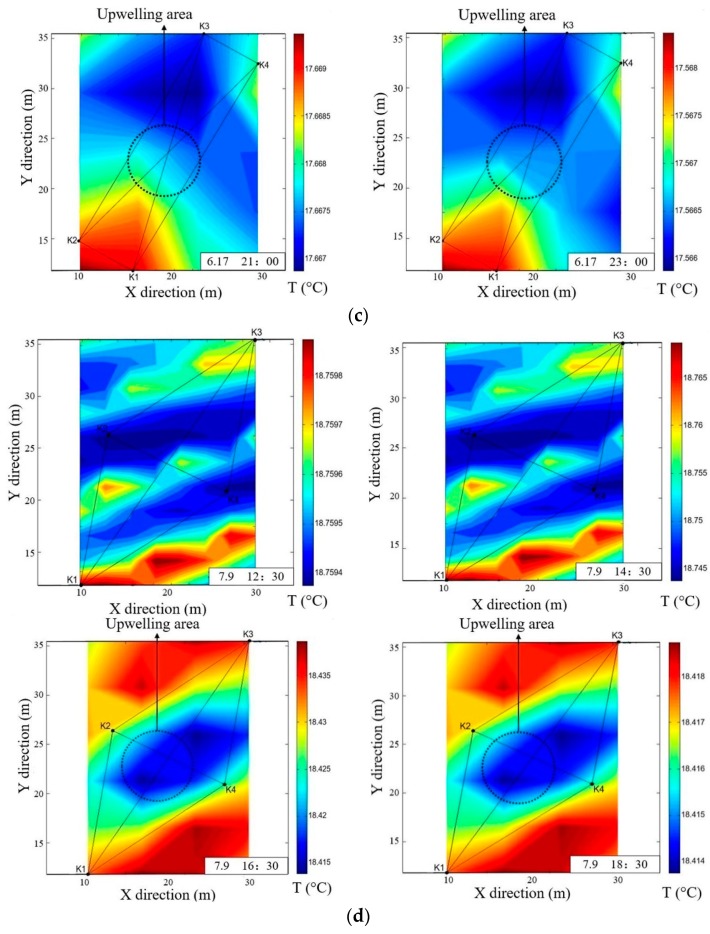
Horizontal mapping. (**a**) Temperature field mapping for right triangle layout; (**b**) Temperature field mapping for isosceles triangle layout; (**c**) Temperature field mapping for rectangular layout; (**d**) Temperature field mapping for lozenge layout.

After obtaining the travel time, we use the inversion method to map the temperature field. The resulting horizontal temperature field for each station layout is displayed in Figure 8. When the upwelling is in progress, its position is marked with a dashed circle in the figure. In each station layout, the results before starting the upwelling and during the upwelling are presented with 2-h intervals. For example, for the right triangle layout, the horizontal temperature field is mapped at 14:00, and 16:00 with no upwelling, and at 18:00 and 20:00 with upwelling occurring. In addition, to prevent the effect of regularities in the station layout, the calculated domain was rotated by 45°. Therefore, the coordinates in Figure 8 represent those after rotating the original width and length direction of the tank.

Based on the results of horizontal temperature field, we can see that:

(1) The horizontal temperature distribution of a certain depth could be obtained and displayed. This depth is decided by the transducer placement. To obtain accurate temperature mapping, we suggest each transducer be placed at the same depth.
(2) The temperature of the upwelling area is significantly lower than that of its surrounding regions; this is obvious in Figure 8a,b,d. This is because the cooler water at the depth of 4 m is brought to the surface by upwelling, reducing the water temperature at the surface of the upwelling area. The lowest water temperature is often at the upwelling center.
(3) It is most obvious in Figure 8d that the temperature in the upwelling area is much lower than its surrounding area. Compared with Figure 8a,b, the number of stations in Figure 8d is 4; compared with Figure 8c, the station spacing of Figure 8d is larger. We conclude that increasing the number of stations and station spacing may help to improve the accuracy of the temperature mapping.
(4) The temperature distribution on Figure 8c did not change much before and after the upwelling, possibly because stations K1 and K2, K3 and K4 are positioned too close to each other, resulting in a non-ideal inversion process. However, due to the noticeable change in average temperature, upwelling phenomenon could still be detected.

### 4.3. Comparison of CAT Results with Temperature Sensor Results

**Figure 9:**
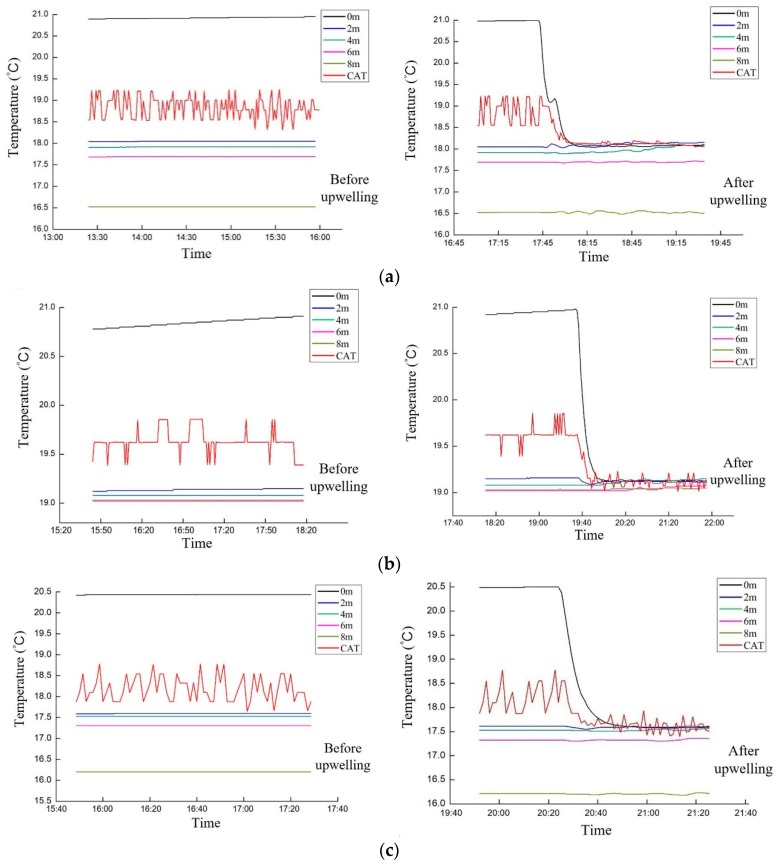

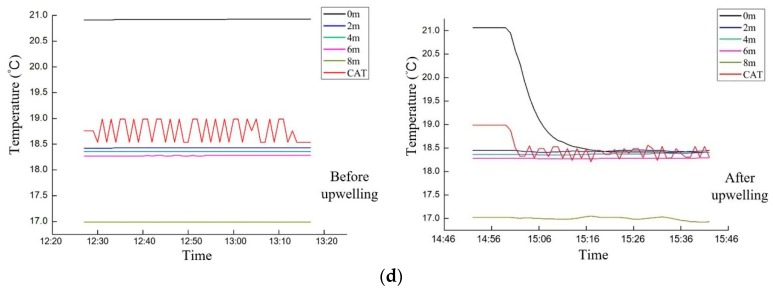
Comparison of CAT results with temperature sensor results. (**a**) The results comparison for right triangle layout; (**b**) The results comparison for isosceles triangle layout; (**c**) The results comparison for rectangular layout; (**d**) The results comparison for lozenge layout.

To verify the CAT observation results, we also measured the water temperature in the vertical direction. First, five temperature sensors named Minilog-II-T were deployed vertically at the depth of 0, 2, 4, 6, and 8 m respectively, to form a temperature chain. The temperature chain was attached to the top of the gas injection frame. Second, the sensors transmit temperature data to the computer via Bluetooth. The observed results are compared with the former obtained CAT results as shown in Figure 9.

Based on the comparison results in Figure 9, we can see that:

(1) All figures in Figure 9 show that the CAT results are distributed between the depth of 0 and 2 m. This is consistent with the deployed depth of transducer, i.e., approximate 1 m.
(2) The water temperature of the anechoic tank decreases from the surface to the bottom. Especially between 0 and 2 m, the temperature drops sharply. This phenomenon indicates that the closer the transducer is placed on the surface, the more accurate temperature changes it could capture.
(3) Figure 9 also shows that the surface temperature is between 20.5 and 21 °C. After upwelling the water at depth of 4m, the surface temperature drops by about 3 °C.
(4) After the upwelling, the CAT observation results are approximate to the water temperature at the surface. Before the upwelling begins, temperature from CAT observation fluctuates and remains within a certain range. After the upwelling, the temperature begins to drop, but stabilizes as the water flow with different depths is mixed. The temperature change captured by CAT before and after the upwelling is approximately 1 °C.
(5) Figure 9d produces the best results in term of the least temperature fluctuating range with 0.5 °C in CAT observation results. This is also consistent with the conclusion from the inversion results in Section 4.2.

### 4.4. The Accuracy Analysis of CAT Observation Results

The accuracy of CAT observation results is affected by the resolution of travel time. The resolution of travel time $t_{r}$ using M sequence is calculated by:

(14) $$t_{r}=\frac{Q}{f}$$

where Q is the cycle-per-digit of M sequence and f is the frequency. The resolution of sound speed deviation $\delta C_{r}$ is expressed by:

(15) $$\delta Cr=\delta C_{e1}=-\frac{{C_{0}}^{2}}{L}t_{r}$$

where *L* is the distance of two stations and $C_{0}$ is the reference sound speed. To further increase observation results accuracy, $\delta C_{r}$ could be recalculated by:

(16) $$\delta Cr=\delta C_{e2}=\frac{\delta C_{e1}}{\sqrt{N}}$$

where N is decided by the signal repeat number and is 8 as mentioned in Section 3. Within the temperature range of 17–21 °C, sound speed and temperature has an approximately linear relationship, i.e., per 1 m/s increase of sound speed means a 0.31 °C increase of water temperature. Therefore, the temperature deviation δT caused by the change of sound speedcould be calculated.

The parameters of resolution calculation mentioned in Equations (14)–(16) are listed in the Table 1. δT is 0.33–1.32 °C when station-to-station distance is 7.5–30 m. This value agrees well with the overall temperature fluctuation range of CAT displayed in Figure 9.

## 5. Conclusions

In this study, we investigate a CAT method to carry out long-term and continuous temperature field observation in the artificial upwelling area in the anechoic tank. Four different CAT layouts were designed and used to observe the temperature in the experiments. The collected temperature data were processed with cross-correlation to obtain travel time. From travel time, we applied an inversion method to map the horizontal temperature field. From the CAT results and the comparison results, we concluded that:

- The change in temperature field could be successfully observed by comparing the horizontal temperature field before and after the upwelling.
- The CAT observation results are basically consistent with the temperature sensor results. This proves the feasibility of CAT for artificial upwelling observation.
- The temperature field in the experimental area has the following characteristics before and after the upwelling. Before the upwelling, the water temperature in the vertical direction changes obviously. After upwelling, the water temperature on the surface decreases rapidly by approximately 3 °C, and stabilizes due tothe exchange of water flow from different depths.

These characteristics are considered an important reference for subsequent upwelling observation in sea experiments. Moreover, there is still space to improve. For example, the intervals for sending acoustic signals is too long currently. This may lead to the temperature field changes remaining not fully captured at the beginning of the upwelling. Furthermore, each station is required to take turns sending signals because simultaneous transmission, and reception was not possible. This may influence the observation accuracy. In the future, we will conduct offshore experiments with larger station spacing and more CAT stations to obtain long-term and continuous temperature field observations in the artificial upwelling area.

## Figures and Tables

**Table 1:** Parameters of resolution calculation.

Q	f	$C_{0}$	L	N	$\delta C_{e2}$	δT
2	50 kHz	1500 m/s	7.5–30 m	8	4.24 m/s	0.33–1.32
